# The Use of a Droplet Collar Accessory Attached to a Portable near Infrared Instrument to Identify Methanol Contamination in Whisky

**DOI:** 10.3390/s23218969

**Published:** 2023-11-04

**Authors:** Adam Kolobaric, Rebecca Orrell-Trigg, Seth Orloff, Vanessa Fraser, James Chapman, Daniel Cozzolino

**Affiliations:** 1 School of Science, RMIT University, Melbourne 3000, Australia; s3385831@student.rmit.edu.au (A.K.); s3486475@student.rmit.edu.au (R.O.-T.); s3720901@student.rmit.edu.au (S.O.); vanessa.fraser@rmit.edu.au (V.F.); 2 Faculty of Science, University of Queensland, Brisbane 4072, Australia; james.chapman@uq.edu.au; 3 Centre for Nutrition and Food Sciences, Queensland Alliance for Agriculture and Food Innovation(QAAFI), University of Queensland, Brisbane 4072, Australia

**Keywords:** alcohol, adulteration, methanol, whisky, infrared

## Abstract

The aim of this study was to evaluate the ability of a droplet collar accessory attached to a portable near-infrared (NIR) instrument to characterize the artificial contamination of methanol in commercial whisky samples. Unadulterated samples (n = 12) were purchased from local bottle shops where adulterated samples were created by adding methanol (99% pure methanol) at six levels (0.5%, 1%, 2%, 3%, 4% and 5% *v*/*v*) to the commercial whisky samples (controls). Samples were analyzed using a drop collar accessory attached to a MicroNIR Onsite instrument (900–1650 nm). Partial least squares (PLS) cross-validation statistics obtained for the prediction of all levels of methanol (from 0 to 5%) addition were considered adequate when the whole adulteration range was used, coefficient of determination in cross-validation (R^2^_cv_: 0.95) and standard error in cross of validation (SECV: 0.35% *v*/*v*). The cross-validation statistics were R^2^_cv_: 0.97, SECV: 0.28% *v*/*v* after the 0.5% and 1% *v*/*v* methanol addition was removed. These results showed the ability of using a new sample presentation attachment to a portable NIR instrument to analyze the adulteration of whisky with methanol. However, the low levels of methanol adulteration (0.5 and 1%) were not well predicted using the NIR method evaluated.

## 1. Introduction

One of the principal risks for the consumer of alcoholic beverages (e.g., gin, vodka, whisky) is the potential ingestion of drinks adulterated with not-known raw ingredients, natural metabolites and products of the fermentation, or by the intentional contamination with foreign and harmful substances (e.g., methanol) [1,2,3]. Methanol is a natural ingredient or product from the fermentation present in small amounts in most alcoholic beverages [1,2,3]. However, to make a greater profit, some illegal suppliers can add adulterants such as methanol into alcoholic beverages as this alcohol is cheap and easily accessible [1,2,3]. Methanol itself is not harmful to humans, but it can be converted into highly toxic formaldehyde and formic acid when metabolized by the organism after ingestion [1,2,3]. Consequently, alcoholic beverages adulterated with methanol might cause serious health issues to humans, including death [3]. The symptomatology of the intoxication of methanol depends on the ingested amount and varies from headache, nausea, vomiting, to blindness and ultimately death [2,3]. For example, human intake of methanol (approx. 10 mL) can cause blindness, where higher concentrations (>30 mL) might lead to death [2]. In recent years, lower-quality commercial whiskeys sold as top-shelf products could not only damage a producer’s reputation and bottom line of profit but also affect human health [1,2,3,4].

The food supply and value chains are vulnerable to issues related to the safety and security of foods. They include a wide range of incidents that are directly connected with food authenticity, adulteration, or fraud [5,6,7,8]. In the literature, different terminology or words have been utilized to define the widespread number of incidents that are affecting the integrity of the food supply and value chains [5,6,7,8]. Some of the terminology used such as food fraud, food adulteration, food crime, and food terrorism are considered under the food safety and security umbrella [5,6,7,8]. The motivation or objectives involving these types of incidents along the food supply and value chain might be different, and they can range from personal revenge, economic gain to ideological objectives [5,6,7,8,9]. 

Recent disruptions in the food supply and value chains (e.g., regional wars, pandemics, floods, droughts) have shown an increased awareness from consumers about issues associated with food safety and security (e.g., authenticity, adulteration, fraud, provenance) [9,10]. In recent years, issues associated with food authenticity have been considered an integral part of both the food safety and security standards [9,10]. Consequently, food authenticity has been considered an essential element that needs to be incorporated in any process that must fulfill key legislation or regulations aiming to protect the food industry and consumers’ health [9,10]. 

Historically, food has been susceptible to different degrees of intentional and unintentional adulteration [11,12]. Natural fungal contamination can occur during transport and storage [13,14], where mixing the spoilt food with fresh food can occur. Other ways of adulteration that can be critical to consumer health are those associated with the substitution of expensive ingredients or products with inferior quality ingredients by addition of additives, the mixing with low-quality products, or the addition of any other type of adulterants [11,12]. Both food adulteration and fraud are words that are frequently applied in the field. Overall, these terminologies have been defined as “a collective term used to encompass the deliberate and intentional substitution, addition, tampering, or misrepresentation of food, food ingredients, or food packaging; or false or misleading statements made about a product, for economic gain” [5,6,7,9,15]. Even though a common definition of food fraud is difficult to establish, there is a universal consensus that food fraud can be considered or defined as any intentional action carried out for financial gain [5,6,7,9,15]. The US Pharmacopeial Convention established that food fraud must be considered as any food ingredient that has a fraudulent addition of non-authentic substances, removal or replacement of authentic substances without the knowledge of the consumer for the exclusive economic gain of the seller [15]. In this context, food adulteration and fraud are closely associated with economically motivated adulteration, intentional adulteration, and any other issues related to food counterfeiting or food fraud [15]. Consequently, assuring the authenticity of food ingredients and products is critical to prevent not only economic fraud in the supply and value chains but also to reduce the negative impact of these issues on both the consumers (e.g., health issues or even death) and the food manufacturing industry (e.g., stakeholders trust, image of the company). 

Several reports can be found in both the scientific literature and in the media that revealed that the marketing and selling of fraudulent commercial whiskies are increasing in the market, causing a greater concern about the potential risk to health of consumers [1,2,3,4]. Therefore, different analytical methods and techniques have been developed and commercially available in routine laboratories to target these issues [1,2,3,4,15,16,17,18,19]. Techniques such as high-performance liquid chromatography (HPLC), gas chromatography (GC), liquid chromatography (LC), and nuclear magnetic resonance (NMR) spectroscopy have been used comprehensively to measure and monitor the occurrence and concentration of methanol in a wide range of alcoholic beverages (e.g., wine, whisky, vodka) [4,15,16,17,18,19]. Nevertheless, these methods are considered expensive, time-consuming, and laborious to be used along the supply and value chain or as rapid quality control methods by the food manufacturing industry [15,16,17,18,19].

Methods based on vibrational spectroscopy [mid-infrared (MIR), near-infrared (NIR), and Raman spectroscopy)] provide an attractive alternative to identify and monitor both adulteration and fraud in different foods due to their simplicity, low cost, and speed of analysis [15,16,17,18,19]. Different researchers have reported the ability of vibrational spectroscopy (e.g., MIR, NIR and Raman spectroscopy) combined with different chemometric and data mining methods (e.g., principal component analysis, cluster and discriminant analysis, neural networks) to detect and quantify the occurrence of methanol in a wide range of alcoholic beverages including spirits, fruit distillates and wine [20,21,22,23,24,25,26,27,28].

The aim of this study was to evaluate the ability of a portable near-infrared instrument coupled with a droplet collar accessory to identify the artificial contamination of methanol to unadulterated commercial whisky samples.

## 2. Materials and Methods

### 2.1. Sampling and Sample Preparation

Unadulterated commercial whiskey samples (n = 12) were purchased from local bottle shops across Melbourne (Victoria, Australia). Consequently, unadulterated whisky samples were obtained from a diverse source of producers (e.g., Canada, Ireland, Scotland, USA) having different alcohol content (35–40% ABV), malt source, aging conditions (e.g. American oak, new oak), hue and color (see Table 1). Immediately after opening the bottle, the adulterated sample set was created by adding methanol (methyl alcohol, UN 1230, 99% pure methanol, Univar, Australia) at six levels (0.5%, 1%, 2%, 3%, 4% and 5% *v*/*v*) to each of the unadulterated commercial whisky samples (control).

### 2.2. Near Infrared Spectra Collection

**Figure 1:**
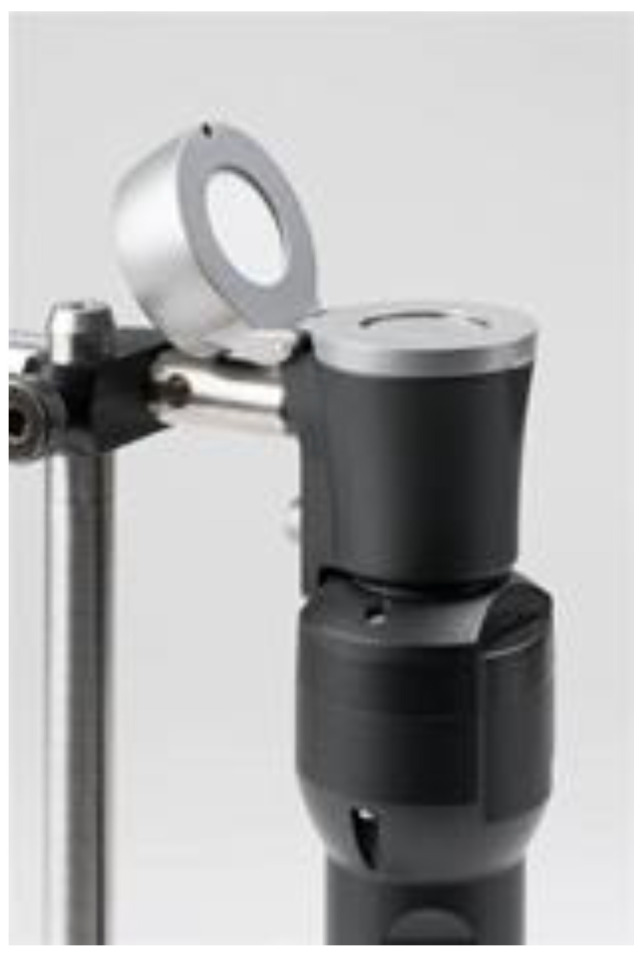
Droplet collar accessory attached to the MicroNIR portable instrument used to analyze the unadulterated and adulterated commercial whisky samples with methanol.

The NIR spectra of the unadulterated commercial whisky samples and adulterated mixtures were randomly collected using a portable NIR instrument (Micro-NIR Onsite, Viavi, Milpitas, CA, USA) operating in the wavelength range between 900 and 1600 nm (10 nm wavelength resolution). The sample presentation method used was the droplet collar accessory shown in Figure 1, provided by the instrument manufacturer. In this sample accessory, 50 µL of the sample (unadulterated and adulterated samples) was pipetted onto the sample window and scanned in triplicate to evaluate the repeatability of the NIR scans. After spectra collection, the MicroNIR droplet collar was cleaned with ethanol then with MilliQ water and dried with a Kimtech® wipes between samples. The spectra collection and instrument setup were controlled using the proprietary software provided by the instrument manufacturer (MicroNIR Prov 3.1, Viavi, Milpitas, CA, USA). The spectral data acquisition parameters were set at 50 ms integration time and averaging 50 scans (MicroNIR Prov 3.1, Viavi, Milpitas, CA, USA) as recommended by the instrument manufacturer. Every 10 samples, a reference spectrum was collected using the Spectralon® tile supplied by the instrument manufacturer. In total, 216 samples were collected (12 commercial labels × 3 replicates × 6 adulteration levels).

### 2.3. Data Analysis

The NIR data were exported in Excel format (*.xls) into The Unscrambler software (version X, CAMO, Oslo, Norway) for data analysis and spectra pre-processing. The NIR spectra were pre-processed using the Savitzky–Golay second derivative (21 smoothing points and second polynomial order) prior to spectra interpretation and chemometric analysis [29]. Principal component analysis (PCA) was used to analyze the data and visualize the effect of the addition of methanol into the unadulterated whisky samples. Leave-one-out cross-validation was applied during the PCA analysis. Partial least squares (PLS) regression models were developed using the levels of methanol adulteration defined in the section above with the NIR region (950 to 1600 nm). In this study, the dataset was divided into two subsets namely calibration and validation, using the Kennard–Stone algorithm [30]. Uniformly distributed 150 samples were selected and used to develop the calibration models, where 66 samples were used for validation. By performing the data partitioning using the Kennard–Stone algorithm, knowledge of the training dataset did not affect the test dataset, and the predictive power of the created model subsequently increased [30]. The coefficient of determination in cross-validation (R^2^_CV_), the standard error in cross-validation (SECV), the standard error in prediction (SEP), bias, slope and residual predictive deviation (RPD = standard deviation / SEP) were used to evaluate the PLS cross-validation models obtained for the prediction of the level of methanol adulteration in the commercial whisky samples analyzed [31,32,33].

## 3. Results and Discussion

### 3.1. Spectra Interpretation 

**Figure 2:**
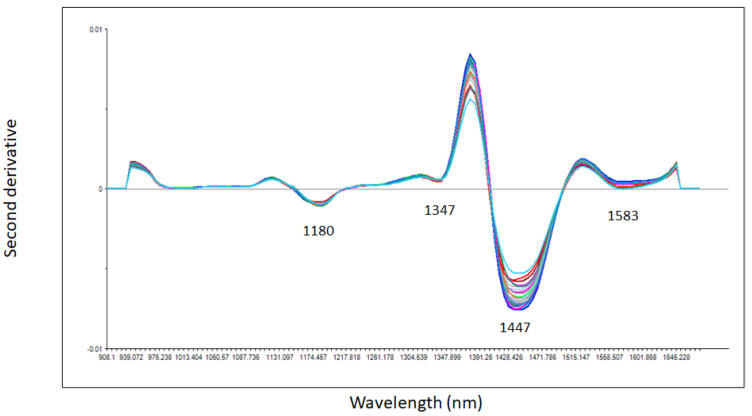
Second derivative near infrared spectra of unadulterated and adulterated commercial whisky samples with methanol analyzed using the droplet collar accessory attached to a portable near infrared instrument.

The second derivative of the NIR spectra of both unadulterated commercial whisky and adulterated mixture samples analyzed using the droplet collar accessory is shown in Figure 2. The second derivative spectra of the samples showed four distinctive absorption bands around 1180 nm associated with O-H stretch second overtone (water and alcohol), around 1347 nm with CH_3_ corresponding with the occurrence of aromatic groups, around 1447 nm with O-H stretch first overtone (water and alcohol) and around 1583 nm associated with the O-H stretch bands of water, alcohol as well as phenolic compounds present in the whisky samples [34,35,36,37,38,39,40,41,42,43,44]. The absorbance band around 1447 nm is also called the dehydration band as reported by other authors [36,37,38]. Specifically, it has been reported that the absorbance value at this specific wavelength can be used to measure low concentrations of methanol in water-methanol mixtures [36,37,38]. However, in this study, slight changes in the absorbance values at 1447 nm were observed as the increase in the concentration of methanol from 0.5% to 5% in the unadulterated and adulterated whiskey samples analyzed.

### 3.2. Principal Component Analysis

**Figure 3:**
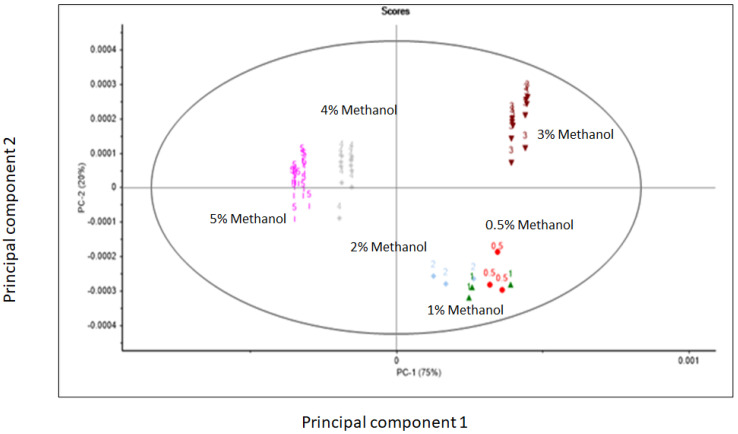
Principal component score plot of unadulterated commercial whisky samples and adulterated samples with methanol, analyzed using the droplet collar attached to a portable near infrared instrument.

Figure 3 shows the principal component score plot of the unadulterated and adulterated commercial whisky samples with different levels of methanol (0.5% to 5% *v*/*v*) and analyzed using the droplet-collar accessory. The first two principal components (PC) explained 95% of the variability in the spectra (PC1: 75% and PC2: 20%). The high concentrations of methanol added to the unadulterated whisky samples were separated along PC1 (control and adulterated high level), while the separation of samples from the low and high concentrations of adulteration was observed along PC2. Overall, the PCA score plot showed that the unadulterated whisky samples (control) were separated from the adulterated mixture samples. The highest loadings derived from the PCA analyzed showed that the main wavelengths contributing to explain the separation along the PC1 were observed around 991 nm associated with O-H bonds derived from alcohol and phenolic compounds [36,42,43,44], at 1162 nm this wavelength can be associated with the O-H stretch second overtones and the occurrence of aromatic groups responsible for the aroma and color of whisky [44], at 1217 nm related with the CH_2_ groups, around 1471 nm associated with the O-H bands related to alcohol while around 1539 nm with the O-H stretch bands of water and alcohol [34,35,36,37,38].

**Figure 4:**
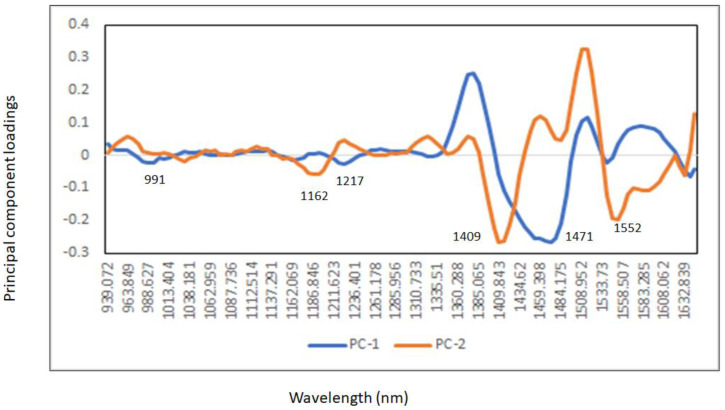
Principal component loadings used by the principal component analysis to evaluate the addition of methanol in the unadulterated commercial whisky samples and analyzed using the droplet collar attached to a portable near infrared instrument.

The highest loadings in PC2 were observed at wavelengths around 1186 nm associated with the C-H bands consistent with aromatic groups present in the whisky samples, around 1409 nm (O-H) and 1477 nm (O-H methanol and O-H hydrogen bonding) while around 1552 nm with O-H or C-H bonds associated with alcohol and phenolic compounds in the whisky samples analyzed [34,35,36,37,38] (Figure 4). It has been also reported that the wavelength region around 1500 nm can be associated with the combination of the O-H and C-H stretch bands originating from alcohols and contributing to the broad O-H overtone band observed in the raw NIR spectra [36,41]. It is important to highlight that absorbance values around 1400 nm might be also associated with the O-H overtones originating from phenolic compounds that are present in both the unadulterated and adulterated whisky samples [36,42,43,44].

### 3.3. Cross Validation Statistics

The PLS cross-validation statistics obtained for the prediction of the level of methanol added to the unadulterated whisky samples in the calibration set using the NIR region (900 to 1600 nm) are shown in Table 2. The PLS cross-validation statistics obtained for the prediction of all levels of methanol addition (from 0% to 5% *v*/*v*) were considered adequate (R^2^_cv_: 0.95, SECV: 0.35% *v*/*v*). However, this model was not able to discriminate between the 0.5% and 1% levels of methanol adulteration. Therefore, it was decided to remove the 0.5% and 1% methanol addition, subsequently, the cross-validation statistics were improved (R^2^_cv_: 0.97, SECV: 0.28% *v*/*v*). During calibration development, the optimal number of latent variables was selected to obtain the lower error in cross-validation to minimize the risk of overfitting (noise or systematic error) and underfitting (missing important information) as reported by other authors [39]. The number of LVs was selected based on the minimization of the SECV, which corresponds to the error acquired during the cross-validation step [39]. Both models were developed using 10 latent variables. These results indicated that NIR spectroscopy might not be able to discriminate or predict satisfactorily the low levels of methanol adulteration in the whisky samples (between 0.5 and 1% *v*/*v*). Similar results were reported by other authors where levels of both ethanol and methanol adulteration were identified in grape derived Pisco distillate samples using different vibrational spectroscopy techniques (e.g., MIR and Raman spectroscopy) [39]. The rapid detection of methanol in a wide range of spirits through the container using a handheld Raman instrument was also reported by other authors in the UK [40]. 

**Figure 5:**
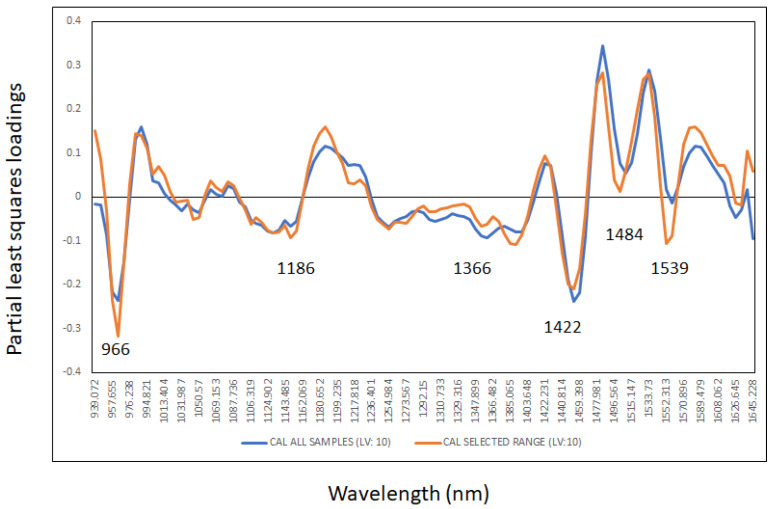
Partial least squares loadings for the optimal models (10 latent variables) used to predict the level of methanol used to adulterate the whisky samples and analyzed using the droplet collar attached to a portable near infrared instrument (range 900 to 1600 nm).

The optimal PLS loadings (10 latent variables) for the cross-validation models were interpreted and reported in Figure 5. Most of the wavelengths used by the PLS models were similar between both models (all samples and samples without including the low concentrations of methanol). The highest PLS loadings in both models were observed around 966 nm, 1186 nm, 1366 nm, 1422 nm, 1484 nm and 1539 nm associated with the same wavelengths and compounds assigned and described in the previous section [33,34,35,36,37]. Additionally, shifts were observed in the loadings at wavelengths around 1422 nm, 1484 nm and 1539 nm due to the addition of methanol (O-H bonds) to the unadulterated whisky samples [33,34,35,36,37].

The prediction statistics using the validation set showed that the SEP obtained were 0.36% *v*/*v* and 0.42% *v*/*v* (see Table 2). The residual prediction deviation (RPD) is a parameter that is used to evaluate the precision of the prediction values in comparison with the average composition of all the samples analyzed [31,33]. It has been stated in the literature that models with an RPD less than 1.5 indicated that the calibration cannot be used, RPD values between 1.5 and 2.0 can be used to differentiate or analyze the variability of the data, RPD values higher than 2.0 indicated good predictive performance of the calibration or validation models where RPD values higher than 3.0 are considered excellent [31,33]. However, the interpretation of the RPD depends on the context and the purpose for which the measurements and predictions will be used [31,33]. The RPD values (SD/SEP) indicated that the calibration models were robust as well as able to predict the range of adulteration with methanol in the set of samples selected as validation (RPD > 4). However, the low levels of methanol adulteration were not well predicted using the NIR method evaluated.

During the last 10 years, an increased number of reports have highlighted the ability of portable infrared instruments to evaluate and predict composition in a wide range of agri-food ingredients and products [45,46,47]. Portable instruments provide valuable advantages compared to laboratory bench instruments that have been extensively evaluated and used in different fields, including foods [45,46,47]. These instruments are portable, lightweight, and easy to use, allowing for the direct and non-destructive measurement of any type of solid sample [45,46,47]. Portable instruments are considered ideal to be utilized along the value chain as they are efficient during the testing process, providing with the knowledge to make informed decisions along the supply and value chain (e.g., from the distillery to the consumer) [45,46,47]. In addition, the direct use of these devices in the supply and value chain will avoid the unnecessary transport of samples to the laboratory, saving time and cost required to evaluate and monitor the adulteration or integrity of the samples [45,46,47]. As stated above, portable instruments have been used to predict the composition and integrity of foods, however, the results of this study showed for the first time the ability of using a new sample presentation attachment (droplet collar) to a portable NIR instrument to analyze the level of adulteration of liquid samples such as whisky. 

## 4. Conclusions 

The adulteration of alcoholic beverages is a global problem having economic and health consequences. This study demonstrated that it was possible to predict the adulteration of methanol in commercial whisky samples using a portable NIR instrument. The implementation of NIR spectroscopy also depends on a better understanding of the spectra of the unadulterated and adulterated samples where the results obtained after the application of chemometrics methods will be used for a rapid analysis, and detection of adulteration in whisky samples. However, low levels of methanol adulteration (0.5 and 1% *v*/*v*) were not well predicted using the method evaluated in this study. Further studies will be carried out to compare the NIR results with those obtained by other methods (e.g., gas chromatography). Finally, it should be noted that the routine application of a calibration model requires continuous validation as it is the critical step to ensure the robustness of the method to predict the levels of adulteration.

## Figures and Tables

**Table 1:** Description of the unadulterated commercial whisky samples analyzed by near infrared spectroscopy and used to develop the classification models.

Brand	ABV%	Colour	Hue	Ageing	Country
Finnlaigh	40	Golden	Dark	n/a	Ireland
Canadian Club	37	Brown	Light	n/a	Canada
Jim Beam Black	40	Brown	Dark	American white oak	USA
Crown Royal	40	Brown	Light	New oak	Canada
Johnny Walker Black Label	40	Brown	Dark	n/a	Scotland
Wild Turkey	43	Brown	Dark	n/a	USA
Jim Beam	37	Brown	Dark	American white oak	USA
Chivas Regal	40	Brown	Dark	n/a	Scotland
Jameson	40	Golden	Dark	Oak	Irish
Johnny Walker Red Label	40	Brown	Dark	n/a	Scotland
Kilbeggan	40	Golden	Dark	n/a	Ireland
Southern Comfort	35	Brown	Light	n/a	USA

**Table 2:** Cross-validation and prediction statistics for the determination of methanol adulteration in commercial whisky samples analyzed using near infrared spectroscopy (900 to 1600 nm).

	N	R^2^_CV_	SECV	Slope	Bias	SEP	RPD	LV
CAL (all samples)	150	0.95	0.35	0.96	0.009			10
CAL (removed 0.5 and 1%)	78	0.97	0.28	0.98	0.005			10
VAL (CAL all samples)	66	0.94		0.94	0.07	0.36	4.4	
VAL (CAL selected samples)	66	0.93		0.94	0.07	0.42	4.16	

CAL: calibration; LV: latent variables; N: number of samples; R^2^_cv_: coefficient of determination in cross validation; RPD: residual predictive value (SD/SEP); SECV: standard error in cross validation; SEP: standard error of prediction; VAL: validation.

## Data Availability

Data is unavailable due to privacy restrictions the data presented in this study are available on request from the corresponding author.
